# Isoniazid Mono-Resistant Tuberculosis: Impact on Treatment Outcome and Survival of Pulmonary Tuberculosis Patients in Southern Mexico 1995-2010

**DOI:** 10.1371/journal.pone.0168955

**Published:** 2016-12-28

**Authors:** Renata Báez-Saldaña, Guadalupe Delgado-Sánchez, Lourdes García-García, Luis Pablo Cruz-Hervert, Marlene Montesinos-Castillo, Leticia Ferreyra-Reyes, Miriam Bobadilla-del-Valle, Sergio Canizales-Quintero, Elizabeth Ferreira-Guerrero, Norma Téllez-Vázquez, Rogelio Montero-Campos, Mercedes Yanes-Lane, Norma Mongua-Rodriguez, Rosa Areli Martínez-Gamboa, José Sifuentes-Osornio, Alfredo Ponce-de-León

**Affiliations:** 1 Centro de Investigación sobre Enfermedades Infecciosas, Instituto Nacional de Salud Pública, Cuernavaca, Morelos, México; 2 Facultad de Medicina, Universidad Nacional Autónoma de México, Ciudad de México, México; 3 Laboratorio de Microbiología, Instituto Nacional de Ciencias Médicas y de Nutrición Salvador Zubirán, Ciudad de México, México; 4 Facultad de Medicina, Universidad Autónoma de San Luis Potosí, San Luis Potosí, San Luis Potosí, México; 5 Dirección Médica, Instituto Nacional de Ciencias Médicas y de Nutrición Salvador Zubirán, Ciudad de México. México; McGill University, CANADA

## Abstract

**Background:**

Isoniazid mono-resistance (IMR) is the most common form of mono-resistance; its world prevalence is estimated to range between 0.0 to 9.5% globally. There is no consensus on how these patients should be treated.

**Objective:**

To describe the impact of IMR tuberculosis (TB) on treatment outcome and survival among pulmonary TB patients treated under programmatic conditions in Orizaba, Veracruz, Mexico.

**Materials and Methods:**

We conducted a prospective cohort study of pulmonary TB patients in Southern Mexico. From 1995 to 2010 patients with acid-fast bacilli or culture proven *Mycobacterium tuberculosis* in sputum samples underwent epidemiological, clinical and microbiological evaluation. We included patients who harbored isoniazid mono-resistant (IMR) strains and patients with strains susceptible to isoniazid, rifampicin, ethambutol and streptomycin. All patients were treated following Mexican TB Program guidelines. We performed annual follow-up to ascertain treatment outcome, recurrence, relapse and mortality.

**Results:**

Between 1995 and 2010 1,243 patients with pulmonary TB were recruited; 902/1,243 (72.57%) had drug susceptibility testing; 716 (79.38%) harbored pan-susceptible and 88 (9.75%) IMR strains. Having any contact with a person with TB (adjusted odds ratio (aOR)) 1.85, 95% Confidence interval (CI) 1.15–2.96) and homelessness (adjusted odds ratio (aOR) 2.76, 95% CI 1.08–6.99) were associated with IMR. IMR patients had a higher probability of failure (adjusted hazard ratio (HR) 12.35, 95% CI 3.38–45.15) and death due to TB among HIV negative patients (aHR 3.30. 95% CI 1.00–10.84). All the models were adjusted for socio-demographic and clinical variables.

**Conclusions:**

The results from our study provide evidence that the standardized treatment schedule with first line drugs in new and previously treated cases with pulmonary TB and IMR produces a high frequency of treatment failure and death due to tuberculosis. We recommend re-evaluating the optimal schedule for patients harboring IMR. It is necessary to strengthen scientific research for the evaluation of alternative treatment schedules in similar settings.

## Introduction

Tuberculosis (TB) is one of the most important infectious diseases worldwide. The World Health Organization (WHO) estimated that during 2015 there were 10.4 million new cases, with a mortality of 1.4 million people;[1] Perhaps one of the most significant factors that impact on control is resistance to first and second line antimicrobials. The resistance among all tuberculosis cases to any drug has ranged from 0% to 70.4%, to isoniazid from 0% to 60.3% and to rifampicin 0% to 44.4%.[2] Isoniazid Mono-Resistance (IMR) is the most common form of mono resistance, and its world prevalence is estimated to range between 0.0 to 9.5% globally (0.0 to 12.8% among new cases and 0.0 to 30.8% among retreated cases).[2]

Burden of TB in Mexico, with 22,294 cases in 2015 (estimated incidence rate of 21, 95 per cent confidence interval (95% CI) 17 to 25 cases per 100,000 inhabitants) and mortality rate of 2.5 (1.8–3.2) per 100,000 inhabitants,[1] entails important loss of potential years of healthy life. Even though several aspects that improved policies and management practices of the country’s National Tuberculosis Prevention and Control Program (e.g. national implementation of WHO’s Directly Observed Therapy–Short course (DOTS) strategy in 1996, creation of the National Tuberculosis Registry, reinforcement of laboratory network and extended availability of first and second line drugs with no cost to patients) have improved since 2000, there are still challenges that hinder TB control. Data from the Mexican Survey on Drug Resistance indicated that in 11.6% of instances disease was caused by strains resistant to a single drug; in 3.5% by strains resistant to one or more drugs (excluding combined resistance to isoniazid and rifampin) and in 2.8% by multidrug resistant (resistant to isoniazid and rifampin) strains. Prevalence of IMR was 3.7% (2.6%-5.1%) overall (3.5% [2.4%-5.1%] among new cases and 6.2% [2.6%-14.0%] among retreated cases).[3]

The WHO standardized schedules have proven to be very efficient in patients with susceptible TB, however, outcomes have been poor when administered to retreated patients, as has been proven in clinical trials[4] and programmatic conditions.[5] Regarding IMR, the more recent WHO recommendations, stated that the evidence available on the treatment of IMR could not address the patient, intervention, comparison and outcome questions[6] and therefore the optimal schedule is still cause for debate. IMR treatment results under established program conditions are variable[7–10] depending on drug resistance prevalence and on whether rifampicin is used throughout treatment.[9]

Since 1995 we have conducted a prospective population-based study in TB patients in Southeast Mexico. We have previously described the clinical consequences and trends of drug resistance.[11, 12] The present study had the purpose of describing clinical outcomes and risk factors of IMR among pulmonary TB patients.

## Methodology

### Study population and enrolment

We conducted a prospective observational cohort study of TB patients as has been previously described. [13, 14] Briefly, the study area includes 12 municipalities in the Orizaba Health Jurisdiction in Veracruz State, Mexico. The study site has an area of 618.11 km^2^ and 413,223 inhabitants, 26.3% of whom live in rural communities.[15]

Between March 1995 to April 2010, we performed passive case findings supported by community health workers and screened persons >15 years old who reported coughing for >15 days. Consenting patients with acid-fast bacilli (AFB) or *Mycobacterium tuberculosis* grown in sputum samples were consecutively recruited over the 15 years and underwent epidemiological, clinical (standardized questionnaire, physical examination, chest radiography, and HIV test), microbiological and molecular evaluations. Chest X-rays were assessed independently by certified radiologists. Staff classifying study outcomes were not blinded, radiologists were blinded to patients’ drug resistance pattern. Personnel were trained in the administration of standardized questionnaires that included previously validated questions. Community health workers ascertained the “homelessness” of participants. We performed cultures on smear-positive sputa from 1995 to 2000; on all sputa (both smear positive and smear negative) from 2000 to 2005; and on sputa from all previously treated TB patients, as well as any new TB patients considered at high risk of having drug resistant TB from 2005 to 2010. Drug susceptibility results were made available to physicians in charge.

Patients received treatment at the local health centers and were followed though the end of their treatment regimen. AFB smears were conducted monthly and at the end of treatment. In the case of patients with smear negative initial results, cultures were conducted at the end of treatment. Treatment was administered at health centers and supervised by health personnel. After treatment was completed, we visited patients’ households annually and administered standardized questionnaires. We collected sputum samples when available to perform smears and MTB cultures, DST and molecular fingerprints to investigate recurrences, relapses, reinfections and vital status, as defined in Table 1.

**Table 1:** Definition of Treatment Outcomes.

Outcomes at the end of treatment	Definition
**Failure**	AFB microscopies or cultures positive at five months or later during treatment.
**Cure**	Treatment completed with disappearance of signs and symptoms and two or more AFB smears or cultures with negative results at the end of therapy.
**Treatment completion**	Completion of treatment without meeting the criteria to be classified as a cure or a failure.
**Death during treatment**	Death due to any cause during therapy.[18]
**Default**	Interruption of treatment for two consecutive months or more.[18]
**Transfer out**	Patient transferred to another institution outside of the study region.
**Outcomes after treatment was completed**	
**Recurrence**	A second or subsequent episode of TB confirmed by AFB smear or culture in a patient with a history of prior treatment.^a^
**Relapse**	TB disease confirmed by AFB smear or culture that occurred after a patient was considered to have completed treatment or to have been cured.[18]
**Reinfections**	Subsequent TB episodes with the same genotype: six or more IS6110 bands in an identical pattern, or < 6 bands with identical IS6110 RFLP patterns and a spoligotype with the same spacer oligonucleotides.
**Deaths after TB treatment was completed**	
**Death due to TB**	Deaths were attributed to TB based on two of the following: death certificate with TB as the main cause of death; interview with a close caregiver who identified TB as a probable cause of death; or positive AFB smear or culture at the time of death.
**Death due to any cause**	Death without specifying cause

^a^ Relapse patients are included within recurrent TB.

Investigators selected and trained the field team composed of physicians, nurses, and field workers who conducted consenting, epidemiological and clinical evaluation and follow-up of patients. A general coordinator supervised field activities and acted as link with the investigators and laboratory team. Periodical meetings were conducted between investigators and field and laboratory teams for monitoring recruitment, clinical activities and follow up.

Following the guidelines of Mexico’s National TB Control Program, between 1995–1998, new cases received two months of isoniazid (H), rifampin (R) pyrazinamide (Z) plus four months of HR (2HRZ/4HR) and retreatment cases also received either ethambutol (E) or streptomycin (S). After 1998, the local health jurisdiction adopted the WHO standard schedule of initiating therapy with 4 drugs (2HRZE/4HR) for newly diagnosed patients and 5 drugs (2HRZES/1HRZE/5HRE) for previously treated patients.[16] The Mexican TB Control Program did not include a specific schedule for MRI. Fluoroquinolones were not included in treatment schedules for these patients.

Treatment was administered under Directly Observed Treatment Short course (DOTS) that included direct and supportive observation (DOT) of drugs by health personnel so as to ensure that prescribed drugs were taken at the right time for the full duration of treatment.[16, 17]

We used the program’s operational definitions for treatment outcomes except for default and death that were defined according to international definitions, Table 1.[16–18]

Rural residence and homelessness were defined as in the Population and Household Census.[19] Usage of alcohol (> ten drinks per week), usage of illegal drugs, (marijuana, cocaine and its derivatives, heroin, methamphetamines, hallucinogens, inhalants and other drugs) were defined as in the National Survey of Addictions (NSA).[20] Patients referring to have “known patients with TB” were defined as having had any contact with patients with TB. Patients were considered to have DM if they had received a previous diagnosis from a physician or oral hypoglycemic medication or insulin administration or treatment.

Body mass index was calculated as weight in kilograms divided by the square of the height in metres (kg/m2).[21] To evaluate health care access, we assessed the distance to the nearest health center and the time elapsed between the onset of symptoms and the beginning of treatment. Molecular fingerprints obtained from first and second or subsequent episodes were compared among recurring and relapsing patients.

### HIV testing

Voluntary HIV testing and counseling was offered to all participants. Results were informed to the patient. In case of positive results he/she was referred to receive appropriate treatment. Testing for HIV was done as per the Mexican HIV Prevention and Control Program using two different tests (UMELISA®HIV 1+2 RECOMBINANT and GENIE FAST HIV Genie™Fast HIV 1/2 BIORAD). All positive results were confirmed by Western blot.[22]

### Mycobacteriology and genotyping

Following the guidelines of Mexico’s National TB Control Program, three sputum samples for each patient were collected at diagnosis, monthly during treatment and when the patient presented with a subsequent episode. We performed Ziehl Neelsen staining, cultures for mycobacteria, species identification, and drug susceptibility testing (DST), following standardized procedures.[23] We used the standard protocol for DST in MGIT 960 (Becton Dickinson Diagnostic instruments, Sparks Md.) followed by the instructions of the manufacturer. The final critical concentrations were 0.1 μg/ml for isoniazid, 1.0 μg/ml for rifampicin, 5.0 μg/ml for ethambutol, and 2.0 μg/ml for streptomycin. BACTEC MGIT 960 DST supplement (0.8 ml) (oleic acid-albumin-dextrose-catalase), 100 μl of the drug stock solution, and 0.5 ml of the suspension containing *M*. *tuberculosis* were added to an MGIT. The GC did not contain any drugs. DST sets were entered into the BACTEC MGIT 960 instrument and continuously monitored until a susceptible or resistant result was obtained. The DST set results were reported by the instrument (determined by the software algorithms, once the GC became positive).[24] Tests were conducted prospectively and results were informed to treatment physicians.

Isolates were genotyped and compared using IS6110-based restriction fragment-length polymorphisms (RFLP) and spoligotyping if the isolate’s IS6110 RFLP patterns had fewer than 6 bands.[25] Patients were considered “clustered” if two or more isolates from different patients were identified within 12 months of each other and had six or more IS6110 bands in an identical pattern, or < 6 bands with identical IS6110 RFLP patterns and a spoligotype with the same spacer oligonucleotides. Cases with a unique genotype pattern (different from all other molecular fingerprints obtained from isolates in the study population) and the first case diagnosed in each cluster likely arose from the reactivation of latent infection caused by *M*. *tuberculosis* strains acquired at a different time or place.[26] Tests were conducted at the Mycobacteriology Laboratory of the Instituto Nacional de Ciencias Médicas y de Nutrición Salvador Zubirán.

### Statistical analysis

For this analysis we included patients who harbored strains susceptible to all tested drugs (isoniazid, rifampicin, ethambutol, and streptomycin) (pansusceptible) (n = 716) and IMR strains (n = 88) totaling 804 patients. We excluded patients with all other types of drug resistance and patients on whom we were unable to perform DST and MDR strains were excluded from all analyses.

We compared characteristics of patients with drug susceptibility testing with those of patients without drug susceptibility testing.

Bivariate and multivariate analyses were performed to assess differences between pan- susceptible and IMR patients. Socio-demographic, clinical, diagnostic, treatment outcome and follow-up characteristics were analyzed.

We analyzed associations between IMR and delayed sputum conversion (after 60 days or more) and treatment failure using multivariate unconditional logistic regression. Variables with p ≤ 0.20 in the bivariate analysis and biological plausibility were included in multivariate models. We estimated the odds ratio (OR) and 95% CI, and identified the covariates that were independently associated with each outcome.

We estimated adjusted hazard ratios (aHR) and 95% CI using Cox proportional hazards models to assess the association of IMR with recurrence, death due to any cause and death due to TB. In the recurrence models, the outcome was the time to diagnosis of recurrence from treatment completion of the previous episode in years. In the mortality models, the outcome was the time to death from diagnosis of the first episode in years. The proportional hazards assumption was verified by introducing terms for the interaction between time and covariates into the model.

By bivariate and multivariate analyses, we compared treatment outcomes among patients stratified by type of patients (new and retreated) and by study period (patients diagnosed between 1995 and 1998, and between 1999 and 2010). All data analysis was performed using STATA 13.1.

### Ethical approval

Participants provided written informed consent to participate in this study. Ethical approval was obtained from the Ethical Commission of the Instituto Nacional de Salud Pública (approval numbers 527). All participants were referred to health facilities to receive treatment in accordance with the stipulations of the National Program for the Prevention and Control of TB.

## Results

Between 1995 and 2010, 1,243 patients older than 15 years were diagnosed with pulmonary TB, of them 902/1,243 (72.57%) had TB drug susceptibility testing. Of patients with DST, 79.38% (716/902) were susceptible to all drugs, 3.22% (29/902) resistant to two drugs (excluding joint resistance to isoniazid and rifampicin); 4.43% (40/902) resistant to both isoniazid and rifampicin and 12.97% (117/902) resistant to a single drug of which 9.75% (88/902) were IMR. For this analysis we included patients who harbored strains susceptible to all drugs (n = 716) and IMR strains (n = 88).

We were unable to culture 27.43% (n = 341) of patients. Reasons included delay in receiving the sample in the laboratory due to remoteness of the patient´s home and consequent low quality of sample. Along the study we implemented strategies to improve sample quality. There were no differences in patients without drug susceptibility test in comparison with patients included in this analysis regarding demographic and epidemiologic characteristics: male gender (56.60% [193/341] vs 57.46% [462/804], p = 0.787), age (42 years, interquartile range [IQR] 29–60,vs 45 years, [IQR 31–58]; p = 0.516), having any contact with a person with TB (44.57% [152/341], vs 43.09% [346/803], p = 0.643), history of previous TB treatment (8.5% [29/341], vs 10.20% [82/804], p = 0.3.75) and homelessness or living in shelters (4.14% [14/338], vs 3.24% [26/803] p = 0.667).

Patients carrying IMR strains were more likely to be homeless [8.0% (7/88) versus 3.1% (22/715), p = 0.021)] and having any contact with a person with TB [54.5% (48/88) versus 41.7% (298/715), p = 0.021]. Proportion of patients having received previous TB treatment was similar between susceptible and IMR patients [9.9% (71/716) versus 12.5% (11/88), p = 0.450], Table 2.

**Table 2:** Socio-demographic, Clinical, and Radiological Characteristics of Patients with Pulmonary Tuberculosis, According to Type of Resistance. Orizaba, Veracruz 1995–2010.

Characteristic	Total	Susceptible	Monoresistant to isoniazid	p-value^a^
	n/N(%)	n/N(%)	n/N(%)	
**Socio-demographic**				
**Age (years), Median (IQR). Number = 804**	46 (31–58)	45(31–59)	45.0(30–55)	0.602^b^
**Males**	462/804 (57.5)	406/716 (56.7)	56/88 (63.6)	0.214
**Less than 6 years of formal schooling**	550/803 (68.5)	484/715 (67.7)	66/88 (75.0)	0.164
**Rural residence**	77/788 (9.8)	68/703 (9.7)	9/85 (10.6)	0.788
**Distance to nearest health center (Meters, median (IQR)]. Number = 803**	705(427–1029)	705(426–1028)	695(443–1074)	0.688 ^b^
**Autochthonous origin**	215/803 (26.8)	194/715 (27.1)	21/88 (23.9)	0.513
**Homelessness or living in shelters**	29/803 (3.6)	22/715 (3.1)	7/88 (8.0)	0.021
**Having any contact with a person with TB**	346/803 (43.1)	298/715 (41.7)	48/88 (54.5)	0.021
**Imprisonment**	86/804 (10.7)	73/716 (10.2)	13/88 (14.8)	0.190
**Access to social security**	286/804 (35.6)	259/716 (36.2)	27/88 (30.7)	0.310
**Usage of illegal drugs**	48/803 (6.0)	41/715 (5.7)	7/88 (8.0)	0.407
**> 10 drinks a week**	198/803 (24.7)	174/715 (24.3)	24/88 (27.3)	0.546
**Diabetes Mellitus**	272/804 (33.8)	243/716 (33.9)	29/88 (33.0)	0.854
**HIV infection**	19/781 (2.4)	18/696 (2.6)	1/85 (1.2)	0.426
**Previous TB treatment**	82/804 (10.2)	71/716 (9.9)	11/88 (12.5)	0.450
**Clinical**				
**Hemoptysis**	269/801 (33.6)	234/714 (32.8)	35/87 (40.2)	0.164
**Fever**	601/801 (75.0)	531/714 (74.4)	70/87 (80.5)	0.215
**Body mass index <20**	336/802 (41.9)	298/714 (41.7)	38/88 (43.2)	0.795
**Radiological**				
**Cavities in chest X ray**	282/718 (39.3)	249/636 (39.2)	33/82 (40.2)	0.849
**More than 10 bacilli per oil immersion field**	198/804 (24.6)	179/716(25.0)	19/88 (21.6)	0.705
**Belongs to a RFLP cluster (IS6110)**	149/759 (19.6)	134/676 (19.8)	15/83 (18.1)	0.484

IQR, Interquartilar range; BMI, Body mass index; HIV, Human immunodeficiency virus; RFLP, Restriction fragment length polymorphism.

^a^ χ2 test.

^b^Mann–Whitney test.

By multivariate analyses, we showed that “homelessness or living in shelters” (Adjusted odds ratio [aOR] 2.76, 95% CI 1.08–6.99) and “having any contact with a person with TB” (aOR1.85,95% CI 1.15–2.96) were associated with IMR adjusting by socio-demographic and clinical variables, Table 3.

**Table 3:** Variables Associated to IMR by Multivariate Analyses.

Variable	Adjusted Odds ratio	95% CI	p-value
**Male**	1.44	0.87–2.37	0.152
**Age**	0.99	0.98–1.01	0.468
**Homelessness or living in shelters**	2.76	1.08–6.99	0.033
**Having any contact with a person with TB**	1.85	1.15–2.96	0.011
**Previous TB treatment**	1.29	0.64–2.59	0.481
**Diabetes mellitus**	1.00	0.58–1.76	0.979
**Cavities in chest X ray**	1.09	0.67–1.76	0.733

TB, Tuberculosis.

Following Mexican treatment guidelines there was no specific treatment for IMR patients. Of the 88 IMR patients, 84 received 3 or 4 drugs during the initial phase (2HRZ [21 patients]; 2HRZE [53 patients]; 3HRZE [9 patients]; or 3HRZ [1 patient]), of which 71 completed the course of six months and 13 were switched to an extended duration regimen as detailed in S1 Table. Among IMR patients, treatment outcomes were similar between patients receiving a 6 month course versus those receiving an extended course.

Patients were followed for an average of 61.7 months (interquartile range [IQR] 26.6 to 97.4). Bivariate Table 4 and multivariate analyses controlled for sociodemographic and clinical variables Table 5 showed that the IMR patients had a higher probability of treatment failure (aHR 12.35, 95% CI (3.38–45.15), p<0.001). Bivariate Table 4 showed that patients with IMR had a significantly greater probability of death due to TB. By Cox adjusted hazards ratios controlled for relevant confounding factors Table 5, we found that the association between IMR and death due to TB occurred only among HIV negative patients (aHR 3.30, (95%CI 1.00–10.84), p<0.05).

**Table 4:** Treatment Outcomes Among Pulmonary Tuberculosis Patients According to Drug Susceptibility. Orizaba, Veracruz, 1995–2010

Characteristic	Total	Susceptible	Monoresistant to isoniazid	p-value^a^
	n/N(%)	n/N(%)	n/N(%)	
**Supervised treatment**	776/784 (99.0)	693/700 (99.0)	83/84 (98.8)	0.870
**AFB conversion >60 days**	212/783 (27.1)	194/697 (27.8)	18/86 (20.9)	0.174
**Time to AFB conversion (days) (n) [Median (IQR)]**	572 [64(57–85)]	516 [64 (57–85)]	56 [67 (59–90)]	0.404^b^
**Time between symptom onset and first AFB (days) (n) [Median (IQR)]**	792 [92(56–168)]	704 [91 (56–164)]	88 [116 (52–225)]	0.174 ^b^
**Time between first AFB and treatment (days) (n) [Median (IQR)]**	757 [6(2–20)]	674 [6 (2–10)]	83 [6 (3–11)]	0.131 ^b^
**Time between symptom onset and treatment (days) (n) [Median (IQR)]**	791 [105(65–178)]	704 [104 (65–172)]	87 [131 (67–243)]	0.122 ^b^
**Treatment result**				
**Cure**	591/804 (73.5)	537/716 (75.0)	54/88 (61.4)	0.006
**Treatment completion**	91/798 (11.4)	81/711 (11.4)	10/88 (11.4)	0.989
**Failure**	10/804 (1.2)	4/716 (0.56)	6/88 (6.8)	<0.001
**Default**	64/804 (8.0)	55/716 (7.7)	9/88 (10.2)	0.405
**Death during treatment**	27/804 (3.4)	21/716 (2.9)	6/88 (6.8)	0.056
**Transfer out**	7/804 (0.9)	6/716 (0.8)	1/88 (1.1)	0.776
**Did not accept treatment**	6/804 (0.7)	5/716 (0.7)	1/88 (1.1)	0.652
**Missing information on outcome**	6/804 (0.7)	5/716 (0.7)	1/88 (1.1)	0.652
**Result after treatment completion**				
**Recurrence**	63/765 (8.2)	54/685 (7.9)	9/80 (11.3)	0.300
**Death due to TB**	27/709 (3.8)	21/639 (3.3)	6/70 (8.6)	0.028
**Death (total)**	208/804 (25.9)	182/716 (25.4)	26/88 (29.5)	0.404

AFB, Sputum smear acid fast bacilli; IQR, Interquartilar range.

^a^χ2 test.

^b^Mann–Whitney test.

**Table 5:** Association of Drug Susceptibility and Selected Clinical Manifestations with Treatment Outcomes Among Patients with Pulmonary TB by Multivariate Analyses.

Variable	Delay in conversion >60 days	Failure	Recurrence	Death due to any cause	Death due to TB (All patients)	Death due to TB (HIV negative patients)
	Odds ratio	Odds ratio	Hazards ratio	Hazards ratio	Hazards ratio	Hazards ratio
	(95% CI)^a^	(95% CI) ^a^	(95% CI) ^b^	(95% CI) ^b^	(95% CI) ^b^	(95% CI) ^b^
	n = 782	n = 803	n = 744	n = 701	n = 701	n = 686
**Mono-resistant to isoniazid (vs pan-susceptible)**	0.68	12.35	1.62	1.33	2.36	3.30
	(0.40–1.19)	(3.38–45.15)^e^	(0.79–3.32)	(0.85–2.09)	(0.76–7.34)	(1.00–10.84) ^c^
**Male**	0.95	1.38	1.46	1.63	1.59	1.45
	(0.66–1.36)	(0.30–6.36)	(0.74–2.88)	(1.12–2.37) ^c^	(0.45–5.54)	(0.44–4.77)
**Age**	0.99	1.00	1.00	1.04	1.01	1.01
	(0.98–0.99)^c^	(0.96–1.04)	(0.99–1.02)	(1.03–1.05) ^e^	(0.98–1.04)	(0.98–1.05)
**>10 drinks a week**	0.72	1.09	2.09	1.90	1.56	—
	(0.47–1.11)	(0.23–5.11)	(1.12–3.92)^c^	(1.33–2.73) ^e^	(0.51–4.82)	—
**Having any contact with a person with TB**	—	—	0.74	0.80	0.77	—
			(0.42–1.28)	(0.59–1.09)	(0.28–2.15)	—
**History of previous TB treatment**	0.85	3.53	1.30	1.01	3.37	3.12
	(0.49–1.46)	(0.83–15.08)	(0.58–2.91)	(0.63–1.63)	(1.02–11.5) ^c^	(0.94–10.38) ^c^
**Diabetes Mellitus**	—	—	1.47	1.59	0.57	0.66
			(0.82–2.62)	(1.17–2.18)^d^	(0.15–2.10)	(0.20–2.16)
**HIV infection**	—	—	4.67	15.26	19.84	—
			(1.10–19.77) ^c^	(7.60–30.64) ^e^	(5.42–72.67) ^e^	—
**Cavities in chest X ray**	—	—	—	1.08	0.96	0.66
	—	—	—	(0.79–1.47)	(0.35–2.64)	(0.20–2.16)

HIV, human immunodeficiency virus; TB, tuberculosis.

^a^ Unconditional logistic regression model.

^b^ Cox proportional hazards model.

^c^ <0.050

^d^<0.010

^e^ <0.001

Patients harboring IMR strains had a greater likelihood of failing treatment when we stratified by study period (1995 to 1998 S2 and S3 Tables and 1999 to 2010 S4 and S5 Tables and type of patients (new S6 and S7 Tables and retreated S8 and S9 Tables). We were unable to obtain a model for the association of IMR and failure among patients diagnosed between 1995 and 1998 since only one patient failed in the group of patients with IMR. By bivariate analysis we found that retreated patients were more likely to die during treatment and to die due to TB after treatment completion S8 Table.

The frequency of recurrence was similar between IMR and pan-susceptible cases (11.3% [9/80] versus 7.9% [54/685] p = 0.300) Table 4. Of the 64 IMR patients who cured or completed treatment, seven relapsed; one of these episodes was documented as a reinfection confirmed with RFLP and spoligotyping, three were documented as the same clone (one developed additional resistance to rifampin in a second episode and to streptomycin in a third episode) and three were no further classified. Of the 618 pansusceptible patients, fifty relapsed after cure or treatment completion. Three were reinfected with a different strain (one with IMR), 33 relapsed with the same clone (one developed mono-resistance to streptomycin) and 14 were no further classified.

## Discussion

In this prospective cohort study conducted in a low HIV prevalence region, we detected high IMR prevalence (9.75%, [88/902]). Risk factors for IMR were having had any contact with a TB patient and homelessness. Our results show that patients harboring IMR strains were more likely to have unfavorable outcomes. As compared to pan-susceptible cases, patients with IMR were more likely to fail treatment. When we stratified by HIV infection, HIV negative patients with IMR were more likely to die, being TB the main cause of death. We did not observe higher frequency of other unfavorable outcomes such as recurrence, relapse or death due to any cause. All of our patients were treated with WHO standardized schedules for new or retreated patients. Mexican treatment guidelines do not include a specific treatment for patients harboring IMR strains.

There is considerable variability in prevalence of IMR in the literature most probably explained by a variety of surveillance methods, prevalence of TB and drug resistance in study populations, treatment regimens, and type of DST, among other reasons. Our results show a prevalence higher than what has been reported in Pakistan, 2.2%[27]; Chile, 2.2%; [28] Madagascar, 3.6%; [29] Taiwan, 5.1%; [8] Denmark, 3.6%; [30] Iran, 6.1%;[31] Israel, 6.4%;[32] Ethiopia, 7.4%; [33] Peru 8.2%;[34] and lower to reports from a tertiary hospital in Taiwan 10.9%.[35] Compared to global estimates, the prevalence of IMR in our study was similar to the upper level of the world estimate of 0.0 to 9.5%.[2] The prevalence in our study was also higher than what was described for Northern Mexico, 4.68% [36] and what was informed in the Mexican drug resistance survey (3.7% [95% CI 2.6%-5.1%]) in 2008–2009[3] suggesting that these figures might be underestimated.

We found that homelessness and having any contact with a patient with TB were associated to IMR. Our results are in agreement with data from previous studies that have revealed that social and biological determinants such as prior tuberculosis treatment;[3, 7, 32] age;[31, 37] smoking or immigration status;[32] illicit drug use;[34] imprisonment, unemployment, drug dealer or commercial sex[37] were associated to IMR. Homelessness has been found to be associated to treatment default which favors emergence of drug resistance[38]. The finding of having had any contact with a patient with TB may indicate that undetected transmission of IMR might be occurring in our study population.

We found that a considerable proportion of patients harboring IMR strains had unfavorable outcomes as compared to patients with susceptible strains. Our results contrast with a study conducted in San Francisco, USA on 137 IMR patients reporting low rates of treatment failure or relapse, 1.7% for patients with IMR treated with 4 or 5 primary drugs not statistically different from 2.2% for pan-susceptible patients.[7] Another study conducted in Denmark also revealed 20% of unfavorable outcomes among 65 IMR patients.[30] Other studies have revealed unfavorable outcomes for IMR patients. In agreement with our study, a study conducted in Taiwan among 425 pulmonary TB patients caused by IMR strains documented unfavorable outcomes, including death, in 14.2% and treatment failure in 2.8%.[8] Other studies conducted in Taiwan [35] and Peru [34] have also reported unfavorable treatment outcomes (14.9% and 25.9%, respectively).

The WHO’s End TB Strategy has proposed that a prerequisite for any national TB programme to reach early diagnosis of TB is a quality-assured laboratory network equipped with rapid diagnostics including the Xpert® MTB/RIF assay [Cepheid, Sunnyvale, CA, United States]) and conducting culture, line probe assay or phenotypic DST, or a combination of these.[39] WHO has recommended that the decisions to scaling up the implementation of these techniques should be made considering the country’s specific epidemiology, the screening strategies used, how to ensure timely access to quality-assured first-line and second-line anti-TB agents, and whether care-delivery mechanisms are appropriate.[40] Mexican TB program officers have taken into consideration Mexico´s low national prevalence of HIV infection (0.2% [0.2–0.3%])[41] MDR prevalence and resource implications, and therefore, have not scaled up to usage of rapid diagnostic tests. Presently the laboratory network includes one central laboratory performing cultures, identification, DST and molecular techniques and 31 referral laboratories performing liquid or solid cultures. Some of these laboratories also conduct DST to first line drugs. Six hundred thirty-eight laboratories distributed all over the country perform AFB smears and collect samples to be referred to culture and DST.[42]

A meta-analysis that evaluated the effects of WHO treatment schedule (2SHRZE/1HRZE/5H_3_R_3_E_3_) for IMR over the rates of failure, relapse and acquired resistance, revealed that in six cohort studies, failure rates were 18%-44% among patients with isoniazid resistance. Among previously treated patients with IMR the combined failure and relapse rates ranged from 0%to over 75%. These authors described that lower failure, relapse and acquired drug resistance rates were associated with longer duration of rifampin, use of streptomycin, daily therapy initially and treatment with a greater number of effective drugs.[9] In our study, the majority of unfavorable outcomes among IMR patients were observed among patients receiving 6 month treatment with 2HRZE/4HR or 2HRZ/4HR. We did not observe differences when treatment was extended to more than 6 months although there were few patients receiving extended treatment. We observed amplification of initial IMR resistance in one patient who relapsed with an MDR strain with the same IS6110 fingerprint as the initial isolate. Several studies have described amplification of resistance in patients prescribed WHO standardized schedule, although amplification of resistance after initial isolation of an IMR strain is infrequent.[43–45] A study conducted in Peru showed that supplementation with a new fluoroquinolone could improve treatment results in patients who were unable to tolerate the continuous use of rifampicin.[34]

We documented that male sex, older age, usage of alcohol, prior TB treatment, diabetes mellitus and HIV infection were covariables independently associated to unfavorable outcomes. Most of these characteristics have been associated to increased failure or death among pulmonary tuberculosis patients in our study area.[14, 15, 46, 47] Few studies have explored covariables associated to unfavorable outcomes when IMR is included as the main independent variable. Comorbidity with cancer and rifampicin interruption [8] and prior TB treatment [35] have been described in two different studies conducted in Taiwan.

Both strengths and weaknesses of this study arise from its extended duration as our study spanned15 years. During this time, two guidelines for treatment of tuberculosis patients were issued in Mexico. The major change was addition of a fourth drug (ethambutol) to the initial phase of treatment. We have therefore stratified our results according to study period. We found that patients harboring IMR strains had an increased likelihood of unfavorable treatment results in both periods. Duration of our study allowed us to find consistent results despite changes in personnel training, patient´s access to timely diagnosis and treatment and other modifications in the health infrastructure that we did not measure. Secondly, since most patients received WHO standard short course chemotherapy, we stratified our patients according to whether they had received prior treatment or were newly diagnosed. We found that patients in all strata had increased likelihood of failure and that retreated patients were more likely to die from TB, although due to small numbers we were only able to conduct bivariate analyses in the group of retreated patients.Thirdly, we were unable to culture and perform DST on all tuberculosis patients diagnosed during the study period. However, we did not find major differences between patients among whom we were able to have DST as compared to those we were unable to study. Fourth, we did not measure adherence to treatment. Finally, we only measured low level resistance and therefore we were unable to identify patients with high level resistance who have been suggested to have better outcomes.[8]

In conclusion, the results of our study provide evidence that new and retreated patients with pulmonary TB harboring IMR strains who are treated with WHO standardized treatment schedule with first line drugs are more likely to suffer unfavorable outcomes as compared to susceptible patients. Different alternatives have been proposed such as enhancement of access to accurate drug sensitivity testing, supplementation with newer fluoroquinolones, extended duration of treatment, early detection of isoniazid resistance and treatment tailoring.[8, 48]

## Supporting Information

S1 Table Treatment Regimens and Outcomes in Isoniazid Mono-resistant. (DOCX)

S2 Table Treatment Outcomes Among Pulmonary Tuberculosis Patients According to Drug Susceptibility. Orizaba, Veracruz, 1995–1998.
(DOCX)

S3 Table Association of Drug Susceptibility with Selected Clinical Manifestations and Treatment Outcomes Among Patients with Pulmonary TB by Multivariate Analyses. Orizaba, Veracruz, 1995–1998.
(DOCX)

S4 Table Treatment Outcomes Among Pulmonary Tuberculosis Patients According to Drug Susceptibility. Orizaba, Veracruz, 1999–2010.
(DOCX)

S5 Table Association of Drug Susceptibility with Selected Clinical Manifestations and Treatment Outcomes Among Patients with Pulmonary TB by Multivariate Analyses. Orizaba, Veracruz, 1999–2010.
(DOCX)

S6 Table Treatment Outcomes Among New Pulmonary Tuberculosis Patients According to Drug Susceptibility. Orizaba, Veracruz, 1995–2010.
(DOCX)

S7 Table Association of Drug Susceptibility with Selected Clinical Manifestations and Treatment Outcomes Among New Patients with Pulmonary TB by Multivariate Analyses. (DOCX)

S8 Table Treatment Outcomes Among Pulmonary Tuberculosis Patients with History of Previous TB Treatment According to Drug Susceptibility. Orizaba, Veracruz, 1995–2010.
(DOCX)

S9 Table Association of Drug Susceptibility with Selected Clinical Manifestations and Treatment Outcomes Among Retreated Patients with Pulmonary TB by Multivariate Analyses. (DOCX)
