# Supplementary material for: Isoniazid Mono-Resistant Tuberculosis: Impact on Treatment Outcome and Survival of Pulmonary Tuberculosis Patients in Southern Mexico 1995-2010
Source: PLoS One. 2016 Dec 28;11(12):e0168955. doi: 10.1371/journal.pone.0168955 (PMC5193431; doi:10.1371/journal.pone.0168955)
Supplement: S1 Table — (DOCX) [file pone.0168955.s001.docx]

**S1 Table. Treatment Regimens and Outcomes in Isoniazid Mono-resistant**

**Cases (N = 88)**

| **Treatment Regimen** | **N (%)** |
| --- | --- |
|  |  |
| 6 months (N= 71) |  |
| 2HRZ/4HR  Cure  Default  Death during treatment  Failure | 18 (25.4%)  11 (15.9%)  3(4.2%)  2 (2.8%)  1(1.4%) |
| Recurrence | 2(2.8%) |
| 2HRZE/4HR  Cure  Treatment completion  Default  Failure  Death during treatment | 49 (69%)  28 (39.4%)  3(4.2%)  4(5.6%)  4 (5.6%)  3 (4.2%) |
| Recurrence | 6 (8.5%) |
| 2HRZE/4HRE  Cure  Default  Failure | 4(5.6%)  2(2.8%)  1 (1.4%) |
| Recurrence | 1 (1.4%) |
| 7-12 months (N=13) |  |
| 2HRZ/5H_2_R_2_  Cure  Treatment completion | 2 (15.4%)  1 (7.7 %)  1 (7.7 %) |
| 3HRZE/6H_3_R_3_  Cure | 1 (7.7 %) |
| 3HRZE/8H_3_R_3_  Treatment completion | 1 (7.7 %) |
| 3HRZ/4H_3_R_3_  Cure | 1 (7.7 %) |
| 3HRZE/4H_3_R_3_ E_6_  Cure | 1 (7.7 %) |
| 3HRZE/5H_3_R_3_E_3_  Treatment completion | 1 (7.7 %) |
| 3HRZE/6H_2_R_2_E  Default | 1 (7.7 %) |
| 3HRZE/6H_2_R_2_E_4_  Cure | 1 (7.7 %) |
| 3HRZE/6H_3_R_3_E_6_  Cure | 1 (7.7 %) |
| 3HRZE/7H_2_R_2_E_6_  Cure | 1 (7.7 %) |
| 3HRZE/8H_3_R_3_E_6_  Cure | 1 (7.7 %) |
| 2HRZ /4HR/X3RZ/1S /1E  Cure | 1(7.7%)  1(7.7%) |
| Unknown (N=4) |  |
| Treatment completion  Death during treatment  Did not accept treatment  Unknown | 1 (25%)  1 (25%)  1 (25%)  1 (25%) |

H, isoniazid; R, rifampicin; E, ethambutol; Z, pyrazinamide;

9 patients suffered a subsequent episode (2 after 2HRZ/4HR, 6 after 2HRZE 4HR, and 1 after 2HRZE/4HRE)
