# Supplementary material for: Isoniazid Mono-Resistant Tuberculosis: Impact on Treatment Outcome and Survival of Pulmonary Tuberculosis Patients in Southern Mexico 1995-2010
Source: PLoS One. 2016 Dec 28;11(12):e0168955. doi: 10.1371/journal.pone.0168955 (PMC5193431; doi:10.1371/journal.pone.0168955)
Supplement: S2 Table — Orizaba, Veracruz, 1995–1998. (DOCX) [file pone.0168955.s002.docx]

**S2 Table. Treatment Outcomes Among Pulmonary Tuberculosis Patients According to Drug Susceptibility. Orizaba, Veracruz, 1995-1998**

| **Characteristic** | **Total** | **Susceptible** | **Monoresistant to isoniazid** | **p-value^a^** |
| --- | --- | --- | --- | --- |
|  | **n/N(%)** | **n/N(%)** | **n/N(%)** |  |
| Self-administered treatment | 4/227 (1.8) | 4/204 (2.0) | 0/23 (0.0) | 0.498 |
| AFB conversion>60 days | 67/227 (29.5) | 57/204 (27.9) | 10/23 (43.5) | 0.121 |
| Time to AFB conversion (days) (n) [Median (IQR)] | 188 [66(57-94)] | 170[66(58-94)] | 18 [60(49-94)] | 0.201^b^ |
| Time between symptom onset and first AFB (days) (n) [Median (IQR)] | 225 [87(51-165)] | 202 [86(53-164)] | 23 [98(38-239)] | 0.794 ^b^ |
| Time between first AFB and treatment (days) (n) [Median (IQR)] | 223 [4(2-8)] | 200 [4(1-7)] | 23 [5(3-8)] | 0.493 ^b^ |
| Time between symptom onset and treatment (days) (n) [Median (IQR)] | 224 [101(60-174)] | 201 [99(60-172)] | 23 [101 (39-243)] | 0.788 ^b^ |
| **Treatment result** |  |  |  |  |
| Cure | 164/230 (71.3) | 149/207 (72.0) | 15/23 (65.2) | 0.496 |
| Treatment completion | 32/230 (13.9) | 30/207 (14.5) | 2/23 (8.7) | 0.446 |
| Failure | 1/230 (0.4) | 0/207 (0.0) | 1/23 (4.3) | 0.003 |
| Default | 22/230 (9.6) | 19/207 (9.2) | 3/23 (13.0) | 0.550 |
| Death during treatment | 7/230 (3.0) | 5/207 (2.4) | 2/23 (8.7) | 0.096 |
| Transfer out | 3/230 (1.3) | 3/207 (1.5) | 0/23 (0.0) | 0.561 |
| Did not accept treatment | 1/230 (0.4) | 1/207 (0.5) | 0/23 (0.0) | 0.738 |
| Missing information on outcome | 0/230 (0.0) | 0/207 (0.0) | 0/23 (0.0) | --- |
| **Result after treatment completion** |  |  |  |  |
| Recurrence | 19/222 (8.6) | 17/201 (8.5) | 2/21 (9.5) | 0.868 |
| Death due to TB | 7/203 (3.4) | 5/184 (2.7) | 2/19 (10.5) | 0.076 |
| Death (total) | 68/230 (29.6) | 60/207 (29.0) | 8/23 (34.8) | 0.563 |

AFB, Sputum smear acid fast bacilli; IQR, Interquartilar range; TB, Tuberculosis.

**^a^**χ2 test.

^b^ Mann–Whitney test.
