# Supplementary material for: Isoniazid Mono-Resistant Tuberculosis: Impact on Treatment Outcome and Survival of Pulmonary Tuberculosis Patients in Southern Mexico 1995-2010
Source: PLoS One. 2016 Dec 28;11(12):e0168955. doi: 10.1371/journal.pone.0168955 (PMC5193431; doi:10.1371/journal.pone.0168955)
Supplement: S3 Table — Orizaba, Veracruz, 1995–1998. (DOCX) [file pone.0168955.s003.docx]

**S3 Table. Association of Drug Susceptibility with Selected Clinical Manifestations and Treatment Outcomes Among Patients with Pulmonary TB by Multivariate Analyses. Orizaba, Veracruz, 1995-1998.**

| Variable | Delay in conversion >60 days | Recurrence HR | Death due to any cause | Death due to TB (All patients)^a^ | Death due to TB (HIV negative patients) ^a^ |
| --- | --- | --- | --- | --- | --- |
|  | (95% CI) ^b^ | (95% CI) ^c^ | (95% CI) ^c^ | (95% CI) ^c^ | (95% CI) ^c^ |
|  | n=226 | n=220 | n= 210 | n= 2010 | n= 206 |
| Mono-resistant to isoniazid (vs pan-susceptible) | 2.23 | 1.07 | 1.24 | 4.87 | 5.58 |
|  | (0.89-5.57) | (0.24-4.74) | (0.55-2.81) | (0.73-32.43) | (0.85-36.77) |
| Male | 1.29 | 1.02 | 0.61 | 0.72 | 1.49 |
|  | (0.65-2.58) | (0.24-4.38) | (0.28-1.36) | (0.05-10.11) | (0.23-9.50) |
| Age | 1.00 | 1.00 | 1.04 | 1.03 | 1.03 |
|  | (0.98-1.02) | (0.97-1.04) | (1.02-1.06)^f^ | (0.97-1.10) | (0.97-1.10) |
| >10 drinks a week | 0.31 | 2.77 | 2.69 | 2.80 | --- |
|  | (0.14-0.71)^e^ | (0.75-10.30) | (1.23-5.87)^d^ | (0.23-33.49) | --- |
| Knows another person with TB | --- | 1.40 | 0.87 | 2.69 | --- |
|  |  | (0.53-3.68) | (0.49-1.54) | (0.42-17.37) | --- |
| History of previous TB treatment | 0.65 | 0.99 | 1.38 | 7.47 | 6.85 |
|  | (0.26-1.64) | (0.22-4.44) | (0.70-2.71) | (1.13-49.10) ^d^ | (1.06-44.06) ^d^ |
| Diabetes Mellitus | --- | 0.88 | 1.17 | --- | --- |
|  |  | (0.25-3.06) | (0.64-2.12) |  |  |
| HIV infection | --- | 10.96 | 53.57 | 101.96 | --- |
|  |  | (1.23-97.72) ^d^ | (15.51-185.05) ^f^ | (5.28-1967.37) ^e^ | --- |
| Cavitation Cavities in chest X ray | --- | --- | 0.78 | 0.40 | 0.55 |
|  | --- | --- | (0.44-1.38) | (0.04-3.72) | (0.06-5.12) |

HIV, human immunodeficiency virus; TB, tuberculosis

^a^ Patients who failed were compared with patients who cured or completed treatment.

^b^ Unconditional logistic regression model.

^c^ Cox proportional hazards model

^d^<0.050

^e^<0.010

^f^<0.001
