# Supplementary material for: Isoniazid Mono-Resistant Tuberculosis: Impact on Treatment Outcome and Survival of Pulmonary Tuberculosis Patients in Southern Mexico 1995-2010
Source: PLoS One. 2016 Dec 28;11(12):e0168955. doi: 10.1371/journal.pone.0168955 (PMC5193431; doi:10.1371/journal.pone.0168955)
Supplement: S4 Table — Orizaba, Veracruz, 1999–2010. (DOCX) [file pone.0168955.s004.docx]

**S4 Table. Treatment Outcomes Among Pulmonary Tuberculosis Patients According to Drug Susceptibility. Orizaba, Veracruz, 1999-2010.**

| **Characteristic** | **Total** | **Susceptible** | **Monoresistant to isoniazid** | **p-value^a^** |
| --- | --- | --- | --- | --- |
|  | **n/N(%)** | **n/N(%)** | **n/N(%)** |  |
| Self-administered treatment | 1/559 (0.2) | 0/497 (0.0) | 1/62 (1.6) | 0.005 |
| AFB conversion>60 days | 145/555 (26.1) | 137/492 (27.8) | 8/63 (12.7) | 0.010 |
| Time to AFB conversion (days) (n) [Median (IQR)] | 384[63(57-80)] | 346[63(57-77)] | 38[70(61-87)] | 0.030^b^ |
| Time between symptom onset and first AFB (days) (n) [Median (IQR)] | 566[93(59-170)] | 501[92(58-164)] | 65[117(60-216)] | 0.170 ^b^ |
| Time between first AFB and treatment (days) (n) [Median (IQR)] | 533[6(3-12)] | 473[6(3-12)] | 60[7(4-12)] | 0.170 ^b^ |
| Time between symptom onset and treatment (days) (n) [Median (IQR)] | 566[106(67-179)] | 502[105(67-171)] | 64[134(67-241)] | 0.110 ^b^ |
| **Treatment result** |  |  |  |  |
| Cure | 427/573 (74.5) | 388/508 (76.4) | 39/65 (60.0) | 0.004 |
| Treatment completion | 59/573 (10.3) | 51/508 (10.0) | 8/65 (12.3) | 0.571 |
| Failure | 9/573 (1.6) | 4/508 (0.8) | 5/65 (7.7) | 0.000 |
| Default | 41/573 (7.2) | 35/508 (6.9) | 6/65 (9.2) | 0.491 |
| Death during treatment | 20/573 (3.5) | 16/508 (3.1) | 4/65 (6.2) | 0.214 |
| Transfer out | 4/573 (0.7) | 3/508 (0.6) | 1/65 (1.5) | 0.387 |
| Did not accept treatment | 5/573 (0.9) | 4/508 (0.8) | 1/65 (1.5) | 0.540 |
| Missing information on outcome | 6/573 (1.0) | 5/508 (1.0) | 1/65 (1.5) | 0.679 |
| **Result after treatment completion** | 44/542 (8.1) | 37/483 (7.7) | 7/59 (11.9) | 0.264 |
| Recurrence |  |  |  |  |
| Death due to TB | 20/506 (4.0) | 16/455 (3.5) | 4/51 (7.8) | 0.133 |
| Death (total) | 139/573 (24.3) | 121/508 (23.8) | 18/65 (27.7) | 0.493 |

AFB, Sputum smear acid fast bacilli; IQR, Interquartilar range; TB, Tuberculosis.

**^a^**χ2 test.

^b^ Mann–Whitney test.
