# Supplementary material for: Isoniazid Mono-Resistant Tuberculosis: Impact on Treatment Outcome and Survival of Pulmonary Tuberculosis Patients in Southern Mexico 1995-2010
Source: PLoS One. 2016 Dec 28;11(12):e0168955. doi: 10.1371/journal.pone.0168955 (PMC5193431; doi:10.1371/journal.pone.0168955)
Supplement: S5 Table — Orizaba, Veracruz, 1999–2010. (DOCX) [file pone.0168955.s005.docx]

**S5 Table. Association of Drug Susceptibility with Selected Clinical Manifestations and Treatment Outcomes Among Patients with Pulmonary TB by Multivariate Analyses. Orizaba, Veracruz, 1999-2010.**

| Variable | Delay in conversion >60 days | Failure^a^ | Recurrence HR | Death due to any cause | Death due to TB (All patients) ^a^ | Death due to TB (HIV negative patients) ^a^ |
| --- | --- | --- | --- | --- | --- | --- |
|  | (95% CI) ^b^ | (95% CI) ^b^ | (95% CI) ^c^ | (95% CI) ^c^ | (95% CI) ^c^ | (95% CI) ^c^ |
|  | n=555 | n= 573 | n=524 | n= 491 | n= 491 | n= 480 |
| Mono-resistant to isoniazid (vs pan-susceptible) | 0.37 | 10.20 | 1.91 | 1.41 | 1.63 | 2.33 |
|  | (0.17-0.81)^d^ | (2.64-39.42)^e^ | (0.83-4.41) | (0.81-2.46) | (0.35-7.68) | (0.46-11.74) |
| Male | 0.88 | 1.18 | 1.46 | 2.41 | 2.45 | 2.29 |
|  | (0.57-1.36) | (0.25-5.53) | (0.66-3.23) | (1.51-3.84)^f^ | (0.46-13.05) | (0.45-11.59) |
| Age | 0.99 | 0.99 | 1.00 | 1.04 | 0.99 | 1.00 |
|  | (0.98-0.99) ^d^ | (0.96-1.04) | (0.98-1.03) | (1.03-1.05) ^f^ | (0.96-1.04) | (0.96-1.05) |
| >10 drinks a week | 0.98 | 0.99 | 2.20 | 2.13 | 1.42 | --- |
|  | (0.58-1.65) | (0.17-5.70) | (1.01-4.76) ^d^ | (1.36-3.35) ^e^ | (0.32-6.38) | --- |
| Knows another person with TB | --- | --- | 1.59 | 0.80 | 0.55 | --- |
|  |  |  | (0.29-1.18) | (0.54-1.18) | (0.14-2.23) | --- |
| History of previous TB treatment | 0.93 | 2.71 | 1.63 | 0.81 | 1.20 | 1.17 |
|  | (0.46-1.86) | (0.49-14.83) | (0.62-4.26) | (0.40-1.63) | (0.14-10.14) | (0.14-9.84) |
| Diabetes Mellitus | --- | --- | 1.73 | 1.84 | 0.92 | 1.17 |
|  |  |  | (0.87-3.43) | (1.25-2.71) ^e^ | (0.22-3.86) | (0.26-5.30) |
| HIV infection | --- | --- | 2.83 | 9.53 | 15.51 | --- |
|  |  |  | (0.37-21.70) | (3.86-23.55) ^f^ | (3.10-77.74) ^e^ | --- |
| Cavitation Cavities in chest X ray | --- | --- | --- | 1.10 | 1.52 | 0.83 |
|  | --- | --- | --- | (0.74-1.62) | (0.44-5.22) | (0.19-3.53) |

HIV, human immunodeficiency virus; TB, tuberculosis

^a^Patients who failed were compared with patients who cured or completed treatment.

^b^ Unconditional logistic regression model.

^c^ Cox proportional hazards model

^d^<0.050

^e^<0.010

^f^<0.001
