# Supplementary material for: Isoniazid Mono-Resistant Tuberculosis: Impact on Treatment Outcome and Survival of Pulmonary Tuberculosis Patients in Southern Mexico 1995-2010
Source: PLoS One. 2016 Dec 28;11(12):e0168955. doi: 10.1371/journal.pone.0168955 (PMC5193431; doi:10.1371/journal.pone.0168955)
Supplement: S6 Table — Orizaba, Veracruz, 1995–2010. (DOCX) [file pone.0168955.s006.docx]

**S6 Table. Treatment Outcomes Among New Pulmonary Tuberculosis Patients According to Drug Susceptibility. Orizaba, Veracruz, 1995-2010**

| **Characteristic** | **Total** | **Susceptible** | **Monoresistant to isoniazid** | **p-value^a^** |
| --- | --- | --- | --- | --- |
|  | **n/N(%)** | **n/N(%)** | **n/N(%)** |  |
| Self-administered treatment | 4/709 (0.6) | 3/634 (0.5) | 1/75 (1.3) | 0.347 |
| AFB conversion>60 days | 193/704 (27.4) | 177/628 (28.2) | 16/76 (21.1) | 0.188 |
| Time to AFB conversion (days) (n) [Median (IQR)] | 516[64(57-84)] | 469[63(57-83)] | 47[64(58-92)] | 0.393^b^ |
| Time between symptom onset and first AFB (days) (n) [Median (IQR)] | 713[93(57-166)] | 636[91(57-164)] | 77[114(60-214)] | 0.190 ^b^ |
| Time between first AFB and treatment (days) (n) [Median (IQR)] | 681[6(2-10)] | 609[5(2-10)] | 72[6(3-10)] | 0.170 ^b^ |
| Time between symptom onset and treatment (days) (n) [Median (IQR)] | 713[106(65-175)] | 637[105(65-171)] | 76[130(67-227)] | 0.150 ^b^ |
| **Treatment result** |  |  |  |  |
| Cure | 541/722 (74.9) | 493/645 (76.4) | 48/77 (62.3) | 0.007 |
| Treatment completion | 79/722 (11.0) | 70/645 (10.9) | 9/77 (11.7) | 0.824 |
| Failure | 7/722 (1.0) | 3/645 (0.5) | 4/77 (5.2) | 0.000 |
| Default | 55/722 (7.6) | 46/645 (7.1) | 9/77 (11.7) | 0.154 |
| Death during treatment | 23/722 (3.2) | 19/645 (2.9) | 4/77 (5.2) | 0.288 |
| Transfer out | 7/722 (1.0) | 6/645 (0.9) | 1/77 (1.3) | 0.755 |
| Did not accept treatment | 4/722 (0.6) | 3/645 (0.5) | 1/77 (1.3) | 0.352 |
| Missing information on outcome | 5/722 (0.7) | 4/645 (0.6) | 1/77 (1.3) | 0.497 |
| **Result after treatment completion** |  |  |  |  |
| Recurrence | 55/690 (8.0) | 47/619 (7.6) | 8/71 (11.3) | 0.279 |
| Death due to TB | 23/643 (3.6) | 19/582 (3.3) | 4/61 (6.6) | 0.188 |
| Death (total) | 185/722 (25.6) | 163/645 (25.3) | 22/77 (28.6) | 0.531 |

AFB, Sputum smear acid fast bacilli; IQR, Interquartilar range; TB, Tuberculosis.

^a^χ2 test.

^b^Mann–Whitney test.
