# Supplementary material for: Isoniazid Mono-Resistant Tuberculosis: Impact on Treatment Outcome and Survival of Pulmonary Tuberculosis Patients in Southern Mexico 1995-2010
Source: PLoS One. 2016 Dec 28;11(12):e0168955. doi: 10.1371/journal.pone.0168955 (PMC5193431; doi:10.1371/journal.pone.0168955)
Supplement: S7 Table — (DOCX) [file pone.0168955.s007.docx]

**S7 Table. Association of Drug Susceptibility with Selected Clinical Manifestations and Treatment Outcomes Among New Patients with Pulmonary TB by Multivariate Analyses.**

| Variable | Delay in conversion >60 days | Failure ^a^ | Recurrence HR | Death due to any cause | Death due to TB (All patients)^a^ | Death due to TB (HIV negative patients)^a^ |
| --- | --- | --- | --- | --- | --- | --- |
|  | (95% CI) ^b^ | (95% CI) ^b^ | (95% CI) ^c^ | (95% CI) ^c^ | (95% CI) ^c^ | (95% CI) ^c^ |
|  | n=703 | n= 821 | n=672 | n= 628 | n= 628 | n= 613 |
| Mono-resistant to isoniazid (vs pan-susceptible) | 0.70 | 12.65 | 1.83 | 1.43 | 1.60 | 2.41 |
|  | (0.37-1.20) | (2.71-59.10)^e^ | (0.85-3.95) | (0.87-2.37) | (0.35-7.36) | (0.49-11.90) |
| Male | 0.86 | 1.81 | 1.37 | 1.83 | 1.95 | 1.56 |
|  | (0.58-1.26) | (0.30-11.01) | (0.65-2.88) | (1.22-2.75) ^d^ | (0.47-8.13) | (0.38-6.37) |
| Age | 0.99 | 1.03 | 1.00 | 1.04 | 1.00 | 1.01 |
|  | (0.98-1.00) | (0.98-1.07) | (0.99-1.02) | (1.03-1.05)^f^ | (.0.97-1.04) | (0.97-1.05) |
| >10 drinks a week | 0.86 | 0.96 | 2.54 | 1.98 | 1.16 | --- |
|  | (0.55-1.35) | (0.15-6.01) | (1.28-5.03) ^e^ | (1.36-2.89) ^f^ | (0.31-4.31) | --- |
| Knows another person with TB | --- | --- | 0.72 | 0.74 | 0.49 | --- |
|  |  |  | (0.40-1.29) | (0.53-1.03) | (0.13-1.86) | --- |
| Diabetes Mellitus | --- | --- | 1.64 | 1.77 | 0.86 | 0.94 |
|  |  |  | (0.88-3.05) | (1.27-2.46) ^e^ | (0.21-3.47) | (0.22-4.09) |
| HIV infection | --- | --- | 1.80 | 15.00 | 16.94 | --- |
|  |  |  | (1.13-20.42)^d^ | (7.42-30.31) ^f^ | (4.40-65.24) ^f^ | --- |
| Cavitation Cavities in chest X ray | --- | --- | --- | 1.04 | 1.19 | 0.77 |
|  | --- | --- | --- | (0.74-1.45) | (0.38-3.69) | (0.19-3.14) |

HIV, human immunodeficiency virus; TB, tuberculosis

^a^Patients who failed were compared with patients who cured or completed treatment.

^b^Unconditional logistic regression model.

^c^ Cox proportional hazards model

^d^<0.050

^e^<0.010

^f^<0.001
