# Supplementary material for: Isoniazid Mono-Resistant Tuberculosis: Impact on Treatment Outcome and Survival of Pulmonary Tuberculosis Patients in Southern Mexico 1995-2010
Source: PLoS One. 2016 Dec 28;11(12):e0168955. doi: 10.1371/journal.pone.0168955 (PMC5193431; doi:10.1371/journal.pone.0168955)
Supplement: S8 Table — Orizaba, Veracruz, 1995–2010. (DOCX) [file pone.0168955.s008.docx]

**S8 Table. Treatment Outcomes Among Pulmonary Tuberculosis Patients with History of Previous TB Treatment According to Drug Susceptibility. Orizaba, Veracruz, 1995-2010**

| **Characteristic** | **Total** | **Susceptible** | **Monoresistant to isoniazid** | **p-value^a^** |
| --- | --- | --- | --- | --- |
|  | **n/N(%)** | **n/N(%)** | **n/N(%)** | **P** |
| Self-administered treatment | 1/77 (1.3) | 1/67 (1.5) | 0/10 (0.0) | 0.697 |
| AFB conversion>60 days | 19/79 (24.1) | 17/69 (24.6) | 2/10 (20.0) | 0.748 |
| Time to AFB conversion (days) (n) [Median (IQR)] | 56[67(58-93)] | 47[67(58-99)] | 9[69(61-76)] | 0.991^b^ |
| Time between symptom onset and first AFB (days) (n) [Median (IQR)] | 79[86(41-195)] | 68[84(43-161)] | 11[155(25-269)] | 0.865 ^b^ |
| Time between first AFB and treatment (days) (n) [Median (IQR)] | 76[7(2-12)] | 65[7(2-12)] | 11[8(3-26)] | 0.575 ^b^ |
| Time between symptom onset and treatment (days) (n) [Median (IQR)] | 78[97(54-200)] | 67[96(60-178)] | 11[158(39-295)] | 0.610 ^b^ |
| **Treatment result** |  |  |  |  |
| Cure | 50/82 (61.0) | 44/71 (62.0) | 6/11 (54.5) | 0.638 |
| Treatment completion | 12/82 (14.6) | 11/71 (15.5) | 1/11 (9.1) | 0.576 |
| Failure | 3/82 (3.7) | 1/71 (1.4) | 2/11 (18.2) | 0.006 |
| Default | 9/82 (11.0) | 9/71 (12.7) | 0/11 (0.0) | 0.211 |
| Death during treatment | 4/82 (4.9) | 2/71 (2.8) | 2/11 (18.2) | 0.028 |
| Transfer out | 0/82 (0.0) | 0/71 (0.0) | 0/11 (0.0) | --- |
| Did not accept treatment | 2/82 (2.4) | 2/71 (2.8) | 0/11 (0.0) | 0.573 |
| Missing information on outcome | 1/82 (1.2) | 1/71 (1.4) | 0/11 (0.0) | 0.692 |
| **Result after treatment completion** |  |  |  |  |
| Recurrence | 8/75 (10.7) | 7/66 (10.6) | 1/9 (11.1) | 0.963 |
| Death due to TB | 4/66 (6.1) | 2/57 (3.5) | 2/9 (22.2) | 0.029 |
| Death (total) | 23/82 (28.0) | 19/71 (26.8) | 4/11 (36.4) | 0.509 |

AFB, Sputum smear acid fast bacilli; IQR, Interquartilar range; TB, Tuberculosis.

^a^χ2 test.

^b^Mann–Whitney test
