# Supplementary material for: Isoniazid Mono-Resistant Tuberculosis: Impact on Treatment Outcome and Survival of Pulmonary Tuberculosis Patients in Southern Mexico 1995-2010
Source: PLoS One. 2016 Dec 28;11(12):e0168955. doi: 10.1371/journal.pone.0168955 (PMC5193431; doi:10.1371/journal.pone.0168955)
Supplement: S9 Table — (DOCX) [file pone.0168955.s009.docx]

**S9 Table. Association of Drug Susceptibility with Selected Clinical Manifestations and Treatment Outcomes Among Patients with History of Previous TB Treatment and with Pulmonary TB by Multivariate Analyses.**

| Variable | Delay in conversion >60 days | Failure^a^ | Recurrence HR | Death due to any cause | Death due to TB (All patients) ^a^ |
| --- | --- | --- | --- | --- | --- |
|  | (95% CI) ^b^ | (95% CI) ^b^ | (95% CI) ^c^ | (95% CI) ^c^ | (95% CI) ^c^ |
|  | n=79 | n= 82 | n=76 | n= 77 | n= 77 |
| Mono-resistant to isoniazid (vs pan-susceptible) | 0.82 | 206.92 | 1.02 | 1.13 | 6.64 |
|  | (0.15-4.37) | (1.74-21563.78)^d^ | (0.11-9.23) | (0.34-3.76) | (0.77-57.01) |
| Male | 1.19 | 0.56 | 1.94 | 0.41 | 0.49 |
|  | (0.39-3.66) | (0.01-23.53) | (0.34-11.02) | (0.14-1.19) | (0.02-10.44) |
| Age | 0.99 | 0.83 | 1.00 | 1.06 | 1.04 |
|  | (0.96-1.03) | (0.67-1.02) | (0.94-1.07) | (1.02-1.09)^e^ | (0.95-1.14) |
| >10 drinks a week | --- | 2.76 | 0.44 | 1.70 | 16.16 |
|  |  | (0.05-141.51) | (0.05-4.22) | (0.42-6.85) | (0.84-310.44) |
| Knows another person with TB | --- | --- | 0.46 | 1.30 | 8.13 |
|  |  |  | (0.09-2.49) | (0.49-3.45) | (0.64-103.30) |
| Diabetes Mellitus | --- | --- | 0.79 | 0.63 | --- |
|  |  |  | (0.11-5.66) | (0.23-1.74) |  |
| HIV infection | --- | --- | --- | --- | --- |
|  |  |  |  |  |  |
| Cavitation Cavities in chest X ray | --- | --- | --- | 1.91 | 0.56 |
|  | --- | --- | --- | (0.74-4.90) | (0.05-6..24) |

HIV, human immunodeficiency virus; TB, tuberculosis

^a^ Patients who failed were compared with patients who cured or completed treatment.

^b^ Unconditional logistic regression model.

^c^ Cox proportional hazards model

^d^<0.050

^e^<0.010
